# 2-Amino-3-carb­oxy­pyrazin-1-ium dihydrogen phosphate

**DOI:** 10.1107/S1600536811017521

**Published:** 2011-05-14

**Authors:** Fadila Berrah, Sofiane Bouacida, Thierry Roisnel

**Affiliations:** a Laboratoire de Chimie Appliquée et Technologie des Matériaux LCATM, Université Larbi Ben M’Hidi, 04000 Oum El Bouaghi, Algeria; b Unité de Recherche de Chimie de l’Environnement et Moléculaire Structurale, CHEMS, Faculté des Sciences Exactes, Université Mentouri Constantine 25000, Algeria; c Centre de Difractométrie X, UMR 6226 CNRS Unité Sciences Chimiques de Rennes, Université de Rennes I, 263 Avenue du Général Leclerc, 35042 Rennes, France

## Abstract

In the crystal structure of the title compound, C_5_H_6_N_3_O_2_^+^·H_2_PO_4_^−^, the dihydrogen phosphate anions are linked through short O—H⋯O hydrogen bonds, forming infinite double chains running parallel to the *b* axis. Centrosymetric N—H⋯O hydrogen-bonded cationic dimers form bridges between these chains by means of inter­molecular N—H⋯O and O—H⋯O hydrogen bonds, leading to a two-dimensional network parallel to (100) in which *R*_3_^3^(12), *R*_4_^3^(10) *R*_2_^2^(8) and *C*(4) graph-set motifs are generated. Weak inter­molecular C—H⋯O hydrogen bonds connect these layers, forming a three-dimensional network.

## Related literature

For hybrid compounds based on *N*-heterocycles, see: Akriche & Rzaigui (2007 ▶); Berrah *et al.* (2011*a* ▶,*b* ▶,*c* ▶); Ouakkaf *et al.* (2011 ▶). For related dihydrogenphosphte compounds, see: Lin *et al.* (2009 ▶); Shao *et al.* (2010 ▶). For hydrogen-bond motifs, see: Bernstein *et al.* (1995 ▶); Etter *et al.* (1990 ▶).
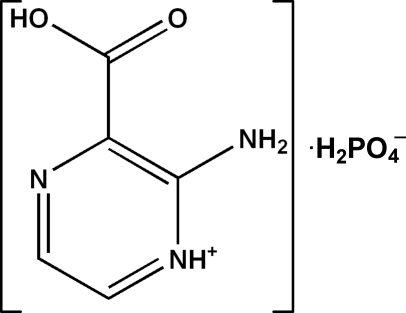


## Experimental

### 

#### Crystal data

C_5_H_6_N_3_O_2_^+^·H_2_PO_4_^−^
*M* *_r_* = 237.11
Monoclinic, 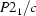

*a* = 8.6076 (5) Å
*b* = 4.6703 (3) Å
*c* = 21.9431 (13) Å
β = 95.573 (2)°
*V* = 877.94 (9) Å^3^
*Z* = 4
Mo *K*α radiation
μ = 0.33 mm^−1^
*T* = 150 K
0.45 × 0.06 × 0.04 mm

#### Data collection

Bruker APEXII diffractometer
Absorption correction: multi-scan (*SADABS*; Sheldrick, 2002 ▶) *T*_min_ = 0.898, *T*_max_ = 0.987
7993 measured reflections
2004 independent reflections
1781 reflections with *I* > 2σ(*I*)
*R*_int_ = 0.025

#### Refinement

*R*[*F* ^2^ > 2σ(*F* ^2^)] = 0.028
*wR*(*F* ^2^) = 0.079
*S* = 1.04
2004 reflections
139 parameters
H-atom parameters constrained
Δρ_max_ = 0.39 e Å^−3^
Δρ_min_ = −0.39 e Å^−3^

### 

Data collection: *APEX2* (Bruker, 2001 ▶); cell refinement: *SAINT* (Bruker, 2001 ▶); data reduction: *SAINT*; program(s) used to solve structure: *SIR2002* (Burla *et al.*, 2005 ▶); program(s) used to refine structure: *SHELXL97* (Sheldrick, 2008 ▶); molecular graphics: *ORTEP-3 for Windows* (Farrugia, 1997 ▶) and *DIAMOND* (Brandenburg & Berndt, 2001 ▶); software used to prepare material for publication: *WinGX* (Farrugia, 1999 ▶).

## Supplementary Material

Crystal structure: contains datablocks global, I. DOI: 10.1107/S1600536811017521/lh5248sup1.cif

Structure factors: contains datablocks I. DOI: 10.1107/S1600536811017521/lh5248Isup2.hkl

Supplementary material file. DOI: 10.1107/S1600536811017521/lh5248Isup3.cml

Additional supplementary materials:  crystallographic information; 3D view; checkCIF report

## Figures and Tables

**Table 1:** Hydrogen-bond geometry (Å, °)

*D*—H⋯*A*	*D*—H	H⋯*A*	*D*⋯*A*	*D*—H⋯*A*
N1—H1*A*⋯O14	0.88	1.94	2.8171 (17)	171
N1—H1*B*⋯O9	0.88	2.09	2.7275 (17)	128
N1—H1*B*⋯O9^i^	0.88	2.37	3.0640 (19)	136
N3—H3⋯O11	0.88	1.79	2.6690 (16)	173
O10—H10⋯O13^ii^	0.84	1.83	2.6591 (16)	169
O12—H12⋯O11^iii^	0.84	1.72	2.5386 (14)	166
O13—H13⋯O14^iv^	0.84	1.64	2.4634 (16)	164
C4—H4⋯O11^v^	0.95	2.43	3.3377 (19)	160

Symmetry codes: (i) 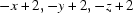; (ii) 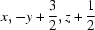; (iii) 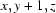; (iv) 
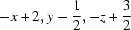
; (v) 
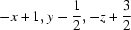
.

**Fig. 1.**
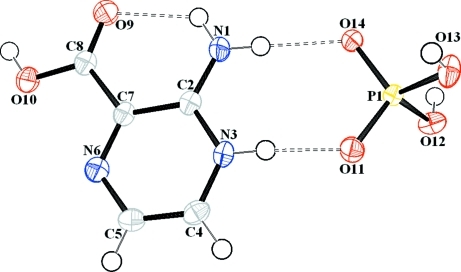
The asymmetric unit of the title compound. Displacement are drawn at the 50% probability level. Hydrogen bonds are shown as dashed lines.

**Fig. 2.**
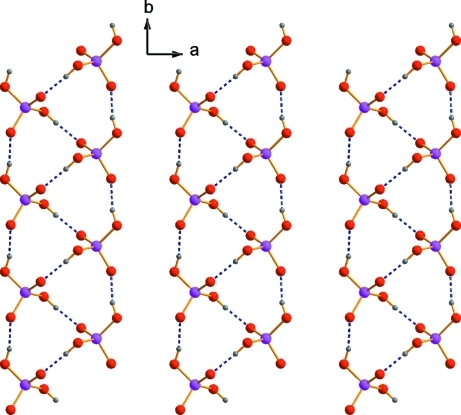
Part of the crystal structure viewed along [001] showing infinite double chains. Hydrogen bonds are shown as dashed lines.

**Fig. 3.**
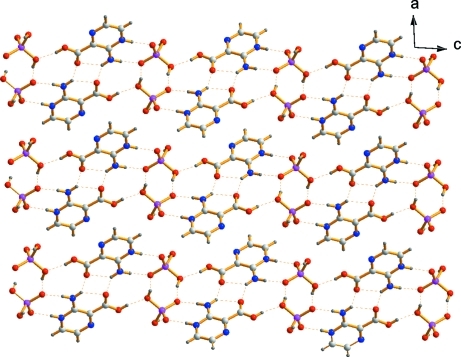
A view parallel to (010) showing cationic dimers and how they link double infinite anionic chains. C—H···O contacts have been omitted for clarity.

**Fig. 4.**
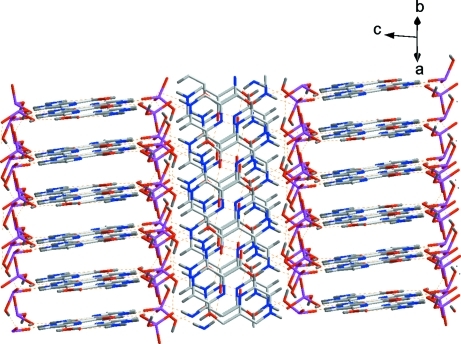
The two-dimensional packing. Hydrogen bonds are shown as dashed lines.

## supplementary crystallographic information

### Comment

In continuation of our search for new hybrids based on protonated N-heterocyclic compounds and inorganic acids we have prepared the title compound. Our recent investigation in this field has revealed the ability of N-heterocyclic derivatives to generate original networks stabilized by hydrogen bonds and has shown how anion substitution may influence the hydrogen-bonding patterns (Berrah *et al.*, 2011*a*,*b*,*c*; Ouakkaf *et al.*, 2011).

The asymmetric unit of the title conpound compound contains one 2-amino-3-carboxypyrazin-1-ium cation and one dihydrogen phosphate anion (Fig. 1). Both entities display geometry similar to that reported in related compounds (Akriche & Rzaigui 2007; Berrah *et al.*, 2011*b*; Shao *et al.*, 2010). dihydrogen phosphate anions linked through strong O—H···O hydrogen bonds (Table 1), form double infinite chains running parallel to the *b* axis (Fig. 2). Similar chains were previously observed in related compounds (Akriche & Rzaigui 2007; Lin *et al.*, 2009). 2-Amino-3-carboxypyrazin-1-ium centrosymetric dimers form bridges between these chains by means of N—H···O and O—H···O hydrogen bonds (Fig. 3) leading to a two-dimensional network (Fig. 4) where *R*^3^_3_(12), *R*^3^_4_(10), *R*^2^_2_(8) and C(4) graph-set motifs are generated (Fig. 2 and Fig. 3)(Etter *et al.*, 1990; Bernstein *et al.*, 1995). Further stabilization is provided by intermolecular C—H···O contacts.

### Experimental

The title compound was synthesized by reacting 3-amino-pyrazine-2-carboxylic acid with phosphoricic acid in a solution of equal volume of H_2_O and CH_3_OH. Slow evaporation leads to well crystallized colourless needles.

### Refinement

H atoms were located in Fourier maps but introduced in calculated positions and treated as riding on their parent atoms (C, N or O) with C—H = 0.95 Å, O—H = 0.84 Å and N—H = 0.88 Å with *U*_iso_(H) = 1.2 *U*_eq_(C or N) and *U*_iso_(H = 1.5 *U*_eq_(O).

### Crystal data

**Table:**

C_5_H_6_N_3_O_2_^+^·H_2_PO_4_^−^	*F*(000) = 488
*M**_r_* = 237.11	*D*_x_ = 1.794 Mg m^−^^3^
Monoclinic, *P*2_1_/*c*	Mo *K*α radiation, λ = 0.71073 Å
Hall symbol: -P 2ybc	Cell parameters from 4062 reflections
*a* = 8.6076 (5) Å	θ = 3.2–27.5°
*b* = 4.6703 (3) Å	µ = 0.33 mm^−^^1^
*c* = 21.9431 (13) Å	*T* = 150 K
β = 95.573 (2)°	Needle, colourless
*V* = 877.94 (9) Å^3^	0.45 × 0.06 × 0.04 mm
*Z* = 4	

### Data collection

**Table:**

Bruker APEXII diffractometer	1781 reflections with *I* > 2σ(*I*)
graphite	*R*_int_ = 0.025
CCD rotation images, thin slices scans	θ_max_ = 27.5°, θ_min_ = 3.2°
Absorption correction: multi-scan (*SADABS*; Sheldrick, 2002)	*h* = −11→7
*T*_min_ = 0.898, *T*_max_ = 0.987	*k* = −6→6
7993 measured reflections	*l* = −28→28
2004 independent reflections	

### Refinement

**Table:**

Refinement on *F*^2^	Primary atom site location: structure-invariant direct methods
Least-squares matrix: full	Secondary atom site location: difference Fourier map
*R*[*F*^2^ > 2σ(*F*^2^)] = 0.028	Hydrogen site location: inferred from neighbouring sites
*wR*(*F*^2^) = 0.079	H-atom parameters constrained
*S* = 1.04	*w* = 1/[σ^2^(*F*_o_^2^) + (0.0383*P*)^2^ + 0.6558*P*] where *P* = (*F*_o_^2^ + 2*F*_c_^2^)/3
2004 reflections	(Δ/σ)_max_ = 0.001
139 parameters	Δρ_max_ = 0.39 e Å^−^^3^
0 restraints	Δρ_min_ = −0.39 e Å^−^^3^

### Special details

**Table:**

Geometry. All e.s.d.'s (except the e.s.d. in the dihedral angle between two l.s. planes) are estimated using the full covariance matrix. The cell e.s.d.'s are taken into account individually in the estimation of e.s.d.'s in distances, angles and torsion angles; correlations between e.s.d.'s in cell parameters are only used when they are defined by crystal symmetry. An approximate (isotropic) treatment of cell e.s.d.'s is used for estimating e.s.d.'s involving l.s. planes.
Refinement. Refinement of *F*^2^ against ALL reflections. The weighted *R*-factor *wR* and goodness of fit *S* are based on *F*^2^, conventional *R*-factors *R* are based on *F*, with *F* set to zero for negative *F*^2^. The threshold expression of *F*^2^ > σ(*F*^2^) is used only for calculating *R*-factors(gt) *etc*. and is not relevant to the choice of reflections for refinement. *R*-factors based on *F*^2^ are statistically about twice as large as those based on *F*, and *R*- factors based on ALL data will be even larger.

### Fractional atomic coordinates and isotropic or equivalent isotropic displacement parameters (Å^2^)

**Table:**

	*x*	*y*	*z*	*U*_iso_*/*U*_eq_	
N1	0.86142 (15)	0.8016 (3)	0.90992 (6)	0.0173 (3)	
H1A	0.8733	0.8741	0.8736	0.021*	
H1B	0.9191	0.8645	0.9425	0.021*	
C2	0.75718 (17)	0.5988 (3)	0.91547 (6)	0.0137 (3)	
N3	0.66953 (15)	0.5047 (3)	0.86483 (5)	0.0149 (3)	
H3	0.6834	0.583	0.8293	0.018*	
C4	0.56229 (17)	0.2967 (3)	0.86666 (7)	0.0165 (3)	
H4	0.5045	0.2339	0.83	0.02*	
C5	0.53675 (17)	0.1756 (3)	0.92166 (7)	0.0171 (3)	
H5	0.4608	0.0288	0.923	0.02*	
N6	0.61838 (15)	0.2626 (3)	0.97404 (6)	0.0166 (3)	
C7	0.72487 (17)	0.4646 (3)	0.97204 (6)	0.0142 (3)	
C8	0.81279 (17)	0.5559 (3)	1.03115 (7)	0.0155 (3)	
O9	0.91059 (13)	0.7446 (2)	1.03400 (5)	0.0214 (3)	
O10	0.77252 (13)	0.4096 (3)	1.07821 (5)	0.0212 (3)	
H10	0.8219	0.4712	1.1104	0.032*	
P1	0.79097 (4)	0.97152 (8)	0.740127 (16)	0.01125 (11)	
O11	0.70388 (12)	0.7004 (2)	0.75265 (5)	0.0161 (2)	
O12	0.66950 (12)	1.1937 (2)	0.71167 (5)	0.0162 (2)	
H12	0.696	1.3588	0.7238	0.024*	
O13	0.89962 (12)	0.9251 (2)	0.68787 (5)	0.0183 (2)	
H13	0.9697	0.8065	0.6994	0.027*	
O14	0.88101 (12)	1.0854 (2)	0.79764 (5)	0.0158 (2)	

### Atomic displacement parameters (Å^2^)

**Table:**

	*U*^11^	*U*^22^	*U*^33^	*U*^12^	*U*^13^	*U*^23^
N1	0.0199 (6)	0.0194 (7)	0.0125 (6)	−0.0029 (5)	0.0013 (5)	0.0017 (5)
C2	0.0139 (7)	0.0139 (7)	0.0134 (6)	0.0041 (5)	0.0019 (5)	−0.0008 (5)
N3	0.0177 (6)	0.0157 (6)	0.0113 (6)	0.0025 (5)	0.0013 (5)	0.0010 (5)
C4	0.0150 (7)	0.0166 (7)	0.0173 (7)	0.0029 (6)	−0.0014 (6)	−0.0021 (6)
C5	0.0141 (7)	0.0177 (7)	0.0193 (7)	−0.0006 (6)	0.0015 (6)	−0.0016 (6)
N6	0.0160 (6)	0.0182 (6)	0.0158 (6)	0.0017 (5)	0.0027 (5)	0.0000 (5)
C7	0.0144 (7)	0.0160 (7)	0.0123 (6)	0.0027 (5)	0.0020 (5)	−0.0003 (5)
C8	0.0160 (7)	0.0172 (7)	0.0135 (7)	0.0028 (6)	0.0023 (5)	−0.0006 (5)
O9	0.0245 (6)	0.0223 (6)	0.0168 (5)	−0.0050 (5)	−0.0003 (4)	−0.0013 (4)
O10	0.0233 (6)	0.0296 (6)	0.0106 (5)	−0.0056 (5)	0.0011 (4)	0.0010 (4)
P1	0.01218 (19)	0.01066 (18)	0.01087 (18)	0.00067 (13)	0.00093 (13)	−0.00036 (13)
O11	0.0213 (5)	0.0112 (5)	0.0156 (5)	−0.0021 (4)	0.0010 (4)	−0.0006 (4)
O12	0.0158 (5)	0.0107 (5)	0.0212 (5)	0.0021 (4)	−0.0025 (4)	−0.0026 (4)
O13	0.0182 (5)	0.0239 (6)	0.0132 (5)	0.0094 (4)	0.0034 (4)	0.0031 (4)
O14	0.0168 (5)	0.0181 (5)	0.0121 (5)	−0.0043 (4)	0.0004 (4)	0.0003 (4)

### Geometric parameters (Å, °)

**Table:**

N1—C2	1.319 (2)	N6—C7	1.319 (2)
N1—H1A	0.88	C7—C8	1.4987 (19)
N1—H1B	0.88	C8—O9	1.2161 (19)
C2—N3	1.3543 (18)	C8—O10	1.3127 (18)
C2—C7	1.442 (2)	O10—H10	0.84
N3—C4	1.343 (2)	P1—O11	1.5101 (11)
N3—H3	0.88	P1—O14	1.5120 (10)
C4—C5	1.370 (2)	P1—O12	1.5597 (11)
C4—H4	0.95	P1—O13	1.5636 (11)
C5—N6	1.3503 (19)	O12—H12	0.84
C5—H5	0.95	O13—H13	0.84
			
C2—N1—H1A	120	N6—C7—C2	122.16 (13)
C2—N1—H1B	120	N6—C7—C8	117.96 (13)
H1A—N1—H1B	120	C2—C7—C8	119.88 (13)
N1—C2—N3	119.16 (13)	O9—C8—O10	124.84 (14)
N1—C2—C7	125.57 (13)	O9—C8—C7	122.65 (14)
N3—C2—C7	115.26 (13)	O10—C8—C7	112.51 (13)
C4—N3—C2	122.68 (13)	C8—O10—H10	109.5
C4—N3—H3	118.7	O11—P1—O14	111.49 (6)
C2—N3—H3	118.7	O11—P1—O12	107.77 (6)
N3—C4—C5	119.62 (14)	O14—P1—O12	111.69 (6)
N3—C4—H4	120.2	O11—P1—O13	111.11 (6)
C5—C4—H4	120.2	O14—P1—O13	111.48 (6)
N6—C5—C4	120.73 (14)	O12—P1—O13	102.94 (6)
N6—C5—H5	119.6	P1—O12—H12	109.5
C4—C5—H5	119.6	P1—O13—H13	109.5
C7—N6—C5	119.53 (13)		
			
N1—C2—N3—C4	179.23 (13)	N3—C2—C7—N6	0.6 (2)
C7—C2—N3—C4	−1.4 (2)	N1—C2—C7—C8	0.5 (2)
C2—N3—C4—C5	1.2 (2)	N3—C2—C7—C8	−178.86 (12)
N3—C4—C5—N6	−0.1 (2)	N6—C7—C8—O9	−178.37 (14)
C4—C5—N6—C7	−0.6 (2)	C2—C7—C8—O9	1.1 (2)
C5—N6—C7—C2	0.4 (2)	N6—C7—C8—O10	1.9 (2)
C5—N6—C7—C8	179.86 (13)	C2—C7—C8—O10	−178.58 (13)
N1—C2—C7—N6	179.93 (14)		

### Hydrogen-bond geometry (Å, °)

**Table:**

*D*—H···*A*	*D*—H	H···*A*	*D*···*A*	*D*—H···*A*
N1—H1A···O14	0.88	1.94	2.8171 (17)	171
N1—H1B···O9	0.88	2.09	2.7275 (17)	128
N1—H1B···O9^i^	0.88	2.37	3.0640 (19)	136
N3—H3···O11	0.88	1.79	2.6690 (16)	173
O10—H10···O13^ii^	0.84	1.83	2.6591 (16)	169
O12—H12···O11^iii^	0.84	1.72	2.5386 (14)	166
O13—H13···O14^iv^	0.84	1.64	2.4634 (16)	164
C4—H4···O11^v^	0.95	2.43	3.3377 (19)	160

Symmetry codes: (i) −*x*+2, −*y*+2, −*z*+2; (ii) *x*, −*y*+3/2, *z*+1/2; (iii) *x*, *y*+1, *z*; (iv) −*x*+2, *y*−1/2, −*z*+3/2; (v) −*x*+1, *y*−1/2, −*z*+3/2.
